# Study protocol of a pragmatic randomized controlled trial incorporated into the Group Lifestyle Balance™ program: the nutrigenomics, overweight/obesity and weight management trial (the NOW trial)

**DOI:** 10.1186/s12889-019-6621-8

**Published:** 2019-03-15

**Authors:** Justine Horne, Jason Gilliland, Colleen O’Connor, Jamie Seabrook, Peter Hannaberg, Janet Madill

**Affiliations:** 1 Health and Rehabilitation Sciences, The University of Western Ontario, London, ON Canada; 2 School of Food and Nutritional Sciences, Brescia University College at The University of Western Ontario, London, ON Canada; 3 The East Elgin Family Health Team, Aylmer, ON Canada; 4 Human Environments Analysis Laboratory, The University of Western Ontario, London, ON Canada; 5 Department of Paediatrics, The University of Western Ontario, London, ON Canada; 6 School of Health Studies, The University of Western Ontario, London, ON Canada; 7 Department of Geography, The University of Western Ontario, London, ON Canada; 8 Lawson Health Research Institute, London, ON Canada; 9 Children’s Health Research Institute, London, ON Canada; 10 Department of Epidemiology and Biostatistics, The University of Western Ontario, London, ON Canada

**Keywords:** Genetics, Lifestyle genomics, Nutrigenomics, Nutrigenetics, Obesity, Overweight, Physical activity, Nutrition

## Abstract

**Background:**

The nutrigenomics, overweight/obesity and weight management trial (NOW Trial) is a pragmatic randomized controlled trial of community-dwelling adults recruited from the Group Lifestyle Balance™ (GLB™) Program. The GLB™ Program (formerly referred to as the Diabetes Prevention Program) is an evidence-based, intensive weight management program, which was offered to overweight/obese patients (BMI ≥ 25.0 kg/m^2^) in a rural Ontario community.

**Methods:**

Patients enrolled in the GLB™ Program were invited to participate in this study. GLB™ groups were randomized 1:1 to receive either the standard GLB™ program + population-based lifestyle advice for weight management, or a modified GLB™ program + personalized, genetic-based lifestyle advice for weight management. The purpose of this study is to determine if the provision of genetic-based lifestyle guidelines is superior to the provision of population-based guidelines in a pragmatic clinical setting to promote changes in: body composition, weight, body mass index, dietary and physical activity habits, as well as attitudes, subjective norms, and behavioural control. The 12-month intervention protocol consists of 23 group-based sessions and 4 one-on-one sessions. Data collection time points include baseline in addition to 3, 6, and 12-month follow up. The comprehensive study design is described in the present manuscript, using both the extended CONSORT checklist for reporting pragmatic trials and the SPIRIT checklist as guidance during manuscript development.

**Discussion:**

Overall, this study seeks to pragmatically determine if the provision of DNA-based lifestyle advice leads to improved health and lifestyle outcomes compared to the provision of standard, population-based lifestyle advice. The results of this trial can be used to inform clinical and community nutrition practice guidelines.

**Trial registration:**

This study was registered with clinicaltrials.gov: NCT03015012 on January 9, 2017.

**Electronic supplementary material:**

The online version of this article (10.1186/s12889-019-6621-8) contains supplementary material, which is available to authorized users.

## Background

Lifestyle modification of nutrition and physical activity are often recommended to help manage overweight and obesity [1]. Despite increased knowledge of beneficial lifestyle strategies for weight management, rates of overweight and obesity continue to climb among adults in Canada and the United States [2, 3]. The Group Lifestyle Balance™ (GLB™) program is one of the most successful lifestyle-based weight management programs and is currently offered in over 80 primary care settings in the United States and is now becoming increasingly prevalent in Canada [4]. The GLB™ program was originally intended only for individuals with prediabetes and was formerly referred to as The Diabetes Prevention Program (DPP). In patients with prediabetes, the DPP lifestyle intervention reduced the risk of progressing to type 2 diabetes by 58%, while the biguanide antihyperglycemic agent, Metformin, reduced the risk of type 2 diabetes by 31% when compared to a placebo pill [5]. Given the documented success of the DPP, the Ontario Ministry of Health and Long-Term Care encouraged program expansion through broader eligibility criteria [6], and as such some clinics are now offering this program for general weight management (regardless of receiving a prediabetes diagnosis), since overweight and obesity are considered risk factors for the development of type 2 diabetes [7].

Although the GLB™ program has proven to be successful [5, 8, 9], a “one-size fits all” approach to weight management has been critiqued by experts, who argue that this generalized approach yields minimal weight loss outcomes that do not satisfy the wants and needs of clinicians, researchers and patients alike [10]. Genetic testing is an innovative tool, which has the potential to facilitate positive lifestyle changes and enhance patient outcomes, though this has been widely debated in the literature in recent years [11–14]. A systematic review found that actionable lifestyle recommendations (e.g., “reduce your consumption of sodium”) facilitated behaviour change greater than the provision of simple genetic-based disease-risk estimates, and that nutrition was the most promising lifestyle habit that could be motivated by lifestyle genomics testing [11].

A few studies have assessed change in weight from the provision of genetic-based information compared to a standard intervention [15–17]. Two studies reported that genetic testing was superior to a standard intervention for changes in weight or BMI [15, 17], and one study showed that adherence to a genetic-based diet was correlated with greater weight loss, whereas adherence to a standard diet was not [16].

There have been considerable scientific advancements in knowledge pertaining to nutrition and physical activity recommendations, which can be personalized based on an individual’s genetic variation. Nutrigenomics is a science that explores the interaction between nutrition, genetics, and health outcomes [18]. The science exploring how nutrition and physical activity, alongside other lifestyle components, can impact health outcomes can be referred to as *lifestyle genomics* [11]. Single nucleotide polymorphisms (SNPs) located within the genes FTO, MC4R, TCF7L2, UCP1, APOA2, and PPARg2 can impact physical activity and dietary approaches to weight management and/or nutritional habits [19–27]. Furthermore, SNPs located within the genes ACTN3, NFIA-AS2, ADRB3, NRF2 and GSTP1 have been shown to impact genetic predisposition to excel in either endurance or strength-based activities [28–32]. These genetic variants were used in the genetic test provided in the present study as they are currently offered through commercial genetic testing [33], thus optimizing the pragmatic nature of this trial.

While genetics certainly plays a role in obesity, there are multiple factors contributing to the current obesity epidemic, including diminished energy expenditure, increased energy intake, rising food costs, the built environment, socioeconomic status, and other social determinants of health [34–39]. Several of these factors can be modified such as energy intake and energy expenditure.

The Theory of Planned Behaviour (TPB) posits that attitudes towards a behaviour, subjective norms, perceived behavioural control and actual behavioural control can be used to predict intentions and behaviours [40]. Although the TPB is one of the most widely-accepted behaviour change theories, it has yet to be incorporated into genetic testing behaviour change research [11, 41] despite a recent call to incorporate this theory into personalized healthcare behaviour change research [41]. By considering this theory, we can account for many contributors impacting behaviour change and therefore account for several confounding factors, which could influence study results. The present randomized controlled trial is the first study to intentionally incorporate the TPB into genetic testing behaviour change research.

The proposed extended CONSORT checklist for reporting pragmatic trials [42] was used to guide the development of the current manuscript. The complete checklist can be reviewed in Additional file 1, with items 1 through 16 being relevant for purposes of this paper.

## Methods/design

### Objectives

The primary objective of this study is to determine if the provision of genetic-based lifestyle advice reduces body fat percentage to a greater extent than the provision of population-based lifestyle advice. Secondary objectives include determining whether the provision of genetic-based lifestyle advice (a) helps to motivate healthful changes to dietary intake and physical activity, (b) leads to greater improvements in anthropometric measures such as weight, BMI, lean mass, fat mass (kg), and water weight, and (c) influences attitudes, subjective norms, behavioural control, and intention to make lifestyle changes. The tertiary objective is to determine if there is a nutrigenomics interaction between ACE rs4343 genetic variation, sodium and water intake, and water weight.

### Hypotheses

Compared to the provision of population-based lifestyle advice, providing DNA-based lifestyle advice will lead to significantly greater improvements in: body fat percentage, attitudes and intentions towards behaviour change, the adoption of healthier dietary and physical activity habits, as well as improved weight, and BMI. Furthermore, we hypothesize that ACE rs4343 genetic variation will lead to increased water weight when sodium intake is high.

### Material and methods

**Fig. 1:**
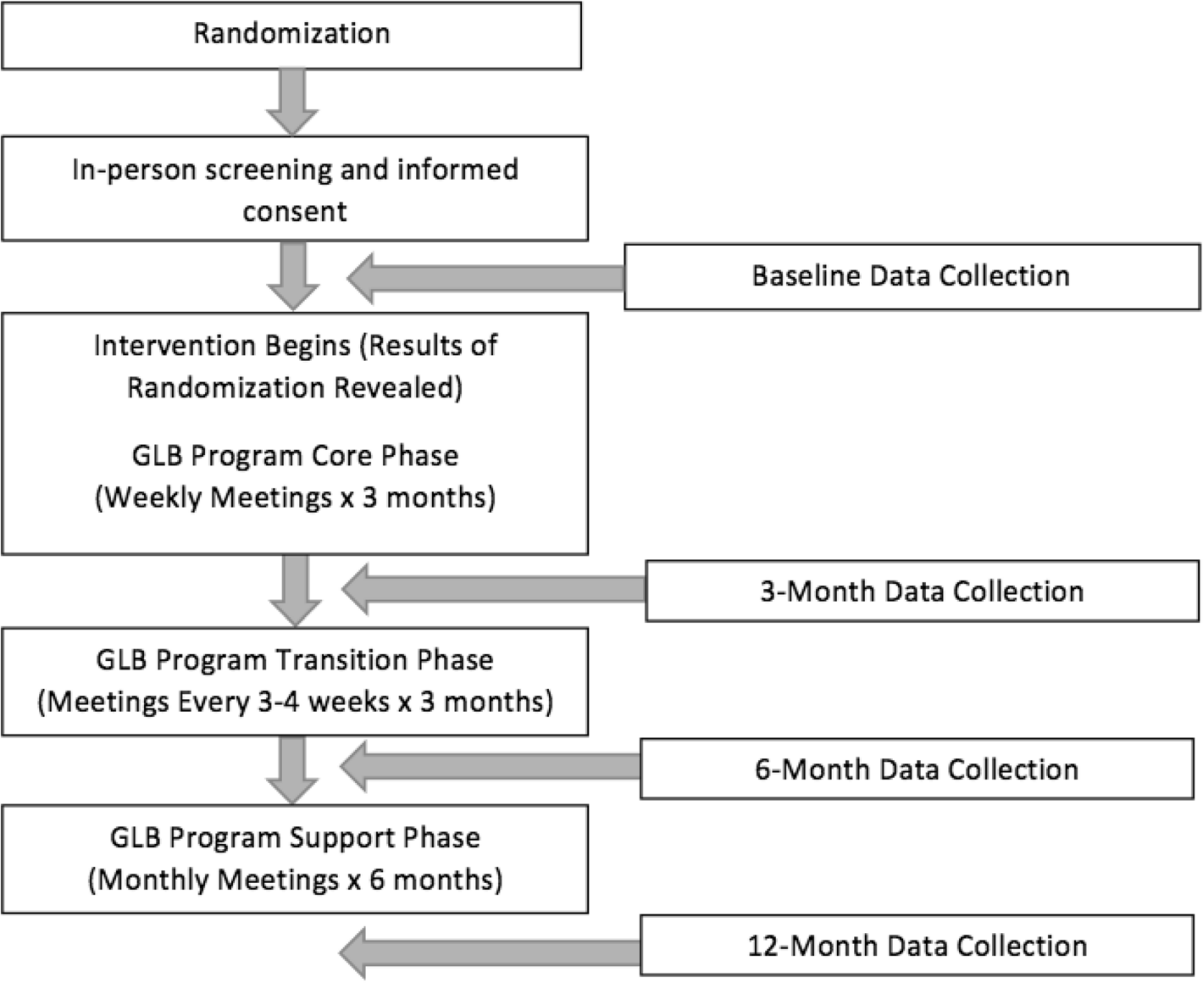
Flow of Study Protocol

**Fig. 2:**
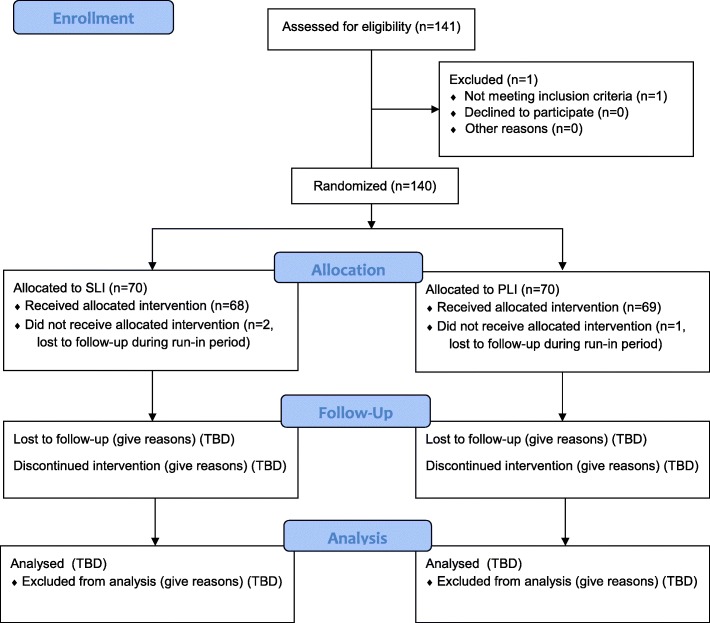
CONSORT 2010 Flow Diagram (Clinical Trial Registration Number: NCT03015012)

**Fig. 3:**
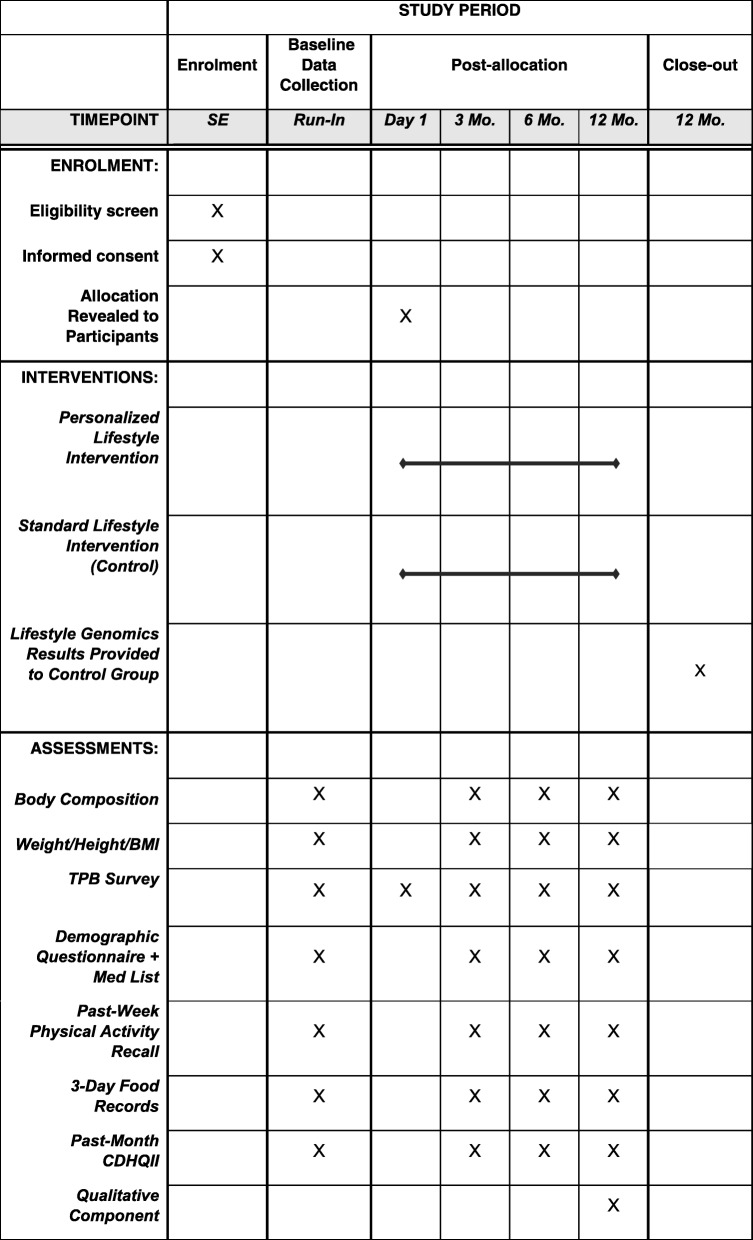
SPIRIT Flow Diagram of The NOW Trial Study Protocol at the EEFHT. SE: study entry; Mo: month

The flow of the study protocol for this parallel group, superiority randomized controlled trial is outlined in Figs. 1, 2 and 3. Further details are provided below.

#### Sample size calculation

Seventy-four participants (*n* = 37 per group) are needed in this study to detect a clinically meaningful difference of 4% in body fat percentage, assuming 80% power, an alpha of 5%, and a standard deviation of 6.1% [43]. We aimed to recruit 88 participants (*n* = 44 per group) to account for the potential dropout rate of 20%. While minimal research exists outlining a clinically meaningful change in body fat percentage, a 5% change in weight (which would come from fat mass, water weight and/or muscle mass) is often reported to be clinically meaningful [44]. Furthermore, clinical experience from the registered dietitians involved in this study helped to determine the clinically meaningful 4% difference mentioned above. This difference has also been supported in published reference standards of body fat percentage in Caucasian adults which indicate that a 4% change in body fat percentage is associated with a 1 – 2 decile change on the reference standards charts [45].

#### Cohort randomization

A cohort randomization model was used rather than subject randomization to allow all participants in a given GLB™ group to obtain the same intervention. At the time of randomization, 12 cohorts (GLB™ groups) were randomized 1:1 to either the personalized lifestyle intervention (PLI) based on genetics, or the standard lifestyle intervention (SLI) based on population-based guidelines. It was anticipated that 12 groups of approximately 7 participants each would be needed to obtain the desired sample size of 88 participants. Prior to obtaining informed consent from participants, randomly permuted blocks were generated using the original generator on an internet-based randomization program [46]. Since participant recruitment was quicker than anticipated and there was an even 1:1 split of a PLI and a SLI group in the last two randomized groups, only 10 of the randomized groups (5 PLI groups, 5 SLI groups) were used, resulting in a total of *N* = 140 participants in the study (mean number of study participants per group ± SD = 14.0 ± 4.1).

#### Recruitment

Participants were recruited from the GLB™ program at the East Elgin Family Health Team (EEFHT). There were two primary methods of recruitment into this program: [1] adults from Elgin and Middlesex Counties in Ontario, Canada were referred to the GLB™ program by healthcare professionals in the area such as registered dietitians (RDs), physicians, nurses, nurse practitioners, and physical therapists; and [2] adults joined the program through word-of-mouth referrals from members of the community. Participants expressing interest in joining the GLB™ program were invited to the EEFHT for an in-person meeting to learn about the NOW Trial, and to provide written, informed consent if they decided to take part in the study. Therefore, participants are highly reflective of typical patients in the GLB™ program. Recruitment occurred from April 2017 until September 2018. This study is registered with clinicaltrials.gov (NCT03015012) and was approved by the Western University Research Ethics Board (108511).

#### Participants: Screening & Informed Consent

Screening and informed consent were completed in person at the EEFHT during the in-person meeting. Inclusion criteria were as follows: BMI ≥ 25.0 kg/m^2^, ≥18 years of age, English-speaking, willing to undergo genetic testing, having access to a computer with internet at least one day per week, and not seeing another healthcare provider for weight loss advice outside of the study. Pregnancy and lactation were considered exclusion criteria.

#### Run-in

Upon obtaining written, informed consent, participants were scheduled for in-person baseline data collection, within approximately 14 days (mean ± SD = 9.3 ± 5.7) prior to the intervention start date. Participants were not given any lifestyle advice during the run-in period.

#### Baseline data collection

All data are entered into the database using unique study codes for each participant and is securely stored in a locked cabinet, in a locked office. Baseline data consisted of a combination of in-person, online, and telephone data collection methods.

Trained research assistants collected 3-day food records over the phone using the validated multiple-pass method [47]. To reduce participant burden, each participant chose to have either 3 separate phone calls (one for each day of intake), or 1 phone call (for all three days of intake). One weekend day and two weekdays were collected. In rare cases where research assistants were unable to reach participants over the phone, the food records were collected in-person at the EEFHT. The food records were then analyzed using the Canadian Nutrient File within the nutritional analysis software program *ESHA Food Processor* (version 11.1).

Participants also completed a self-administered past-month, semi-quantitative, online food frequency questionnaire, the *Canadian Diet History Questionnaire II (CDHQII)*. This questionnaire is a modified version of the *United States Diet History Questionnaire* adapted for Canadian data [48]. Most participants completed this questionnaire away from the EEFHT, but in cases where participants did not have internet access at home (*n* = 3), the CDHQII was self-administered at the EEFHT.

In-person baseline data collection included: measured height and weight (used to calculate BMI), a BIA assessment to obtain body composition data (using the *Body Stat 1500MDD)*, a past-week physical activity recall (used to calculate metabolic equivalents), a baseline demographic questionnaire, a list of medications, and a TPB questionnaire. To optimize reliability, weight and height measurements were taken on the same *Health O Meter Professional* weigh scale and stadiometer, and body composition was assessed using the same BIA machine. The TPB questionnaire was developed based on Ajzen’s Guide to Constructing a TPB Questionnaire [49]. The results for weight, body fat percentage, body fat (kg), lean weight, and water weight from the BIA were communicated to participants during their in-person baseline data collection visit.

To assess possible short-term changes in components of the TPB (e.g. attitudes towards nutrition, physical activity, genetic testing, etc.), the TPB questionnaire was administered twice during the baseline assessment period: once during the one-on-one, in-person meeting (pre-intervention), and once immediately after the first group-based intervention session was delivered (post-intervention).

#### Blinding and allocation concealment

During informed consent meetings, baseline data collection and the run-in period, participants were blinded to their group assignment. However, participants were not blinded to their group assignment during the administration of the second baseline TPB survey (completed immediately after the first group session in order to assess possible changes short-term in key components of the TPB upon receiving either population-based advice or genetic-based advice). Research assistants collecting and analyzing food intake data were also blinded to the study group of the participants and the statistician will be blinded to the group assignments. Since our outcomes included changes in attitudes related to genetic testing for personalized lifestyle advice, as well as change in nutrition and physical activity habits, it was inappropriate to blind the participants throughout the entire duration of the study. Therefore, participants were informed of their group assignment during the first group intervention meeting, as further outlined in section 4.9, below. One author generated the allocation sequence, enrolled participants, facilitated group and one-on-one interventions, collected data, entered data into the database and scheduled participants, and therefore could not be blinded. Allocation was concealed for the other five co-authors.

#### Staggered cohorts

Staggered cohorts have been used to reduce the impact of confounding by indication and have previously been successful in studies comparing active and passive treatment groups [50]. In the present study, staggered cohorts were pre-planned in order to maximize study efficiency and effectiveness. Seasonality and timing of groups were considered in the planning phase to ensure that there was a similar amount of SLI groups and PLI groups offered during the day and evening. Three SLI groups were offered during the day, and 2 SLI groups were offered in the evenings. Likewise, 3 PLI groups were offered during the day, and 2 PLI groups were offered in the evenings. 1 SLI group began in the spring, 2 in the summer, and 2 in the fall. Similarly, 1 PLI group began in the spring, 2 in the summer, 1 in the fall, and 1 in the winter.

#### Interventions

Given its previously documented success [5, 8, 9], the GLB™ program was chosen as the gold standard comparator for this RCT. Furthermore, given that this study is taking place within routine community/clinical practice, it is highly pragmatic with a mean overall PRECIS-2 score of 4.4 (Table 1) [51].

**Table 1:** PRECIS-2 Scoring Tool [51]

PRECIS-2 Domain	Score [Likert scale 1 (very explanatory) - 5 (very pragmatic)]
1. Eligibility: To what extent are the participants in the trial similar to those who would receive this intervention if it was part of usual care?	5
2. Recruitment: How much extra effort is made to recruit participants over and above what would be used in the usual care setting to engage with patients?	5
3. Setting: How different are the settings of the trial from the usual setting?	5
4. Organization: How different are the resources, provider expertise, and the organization of care delivery in the intervention arm of the trial from those available in usual care?	4
5. Flexibility (delivery): How different is the flexibility in how the intervention is delivered and the flexibility anticipated in usual care?	4
6. Flexibility (adherence): How different is the flexibility in how participants are monitored and encouraged to adhere to the intervention from the flexibility anticipated in usual care?	4
7. Follow-up: How different is the intensity of measurement and follow-up of participants in the trial from the typical follow-up in usual care?	3
8. Primary outcome: To what extent is the trial’s primary outcome directly relevant to participants?	5
9. Primary analysis: To what extent are all data included in the analysis of the primary outcome?	TBD
Mean score:	**4.4**

Participants joined the GLB™ group session that best suited their availability, and were blinded to the group intervention assignment at this time. As previously detailed, groups were pre-randomized 1:1 to receive either the standard 12-month GLB™ Program curriculum + a summary report of population-based lifestyle recommendations (SLI/Control Group), or a modified 12-month GLB™ Program + a summary report of DNA-based lifestyle recommendations (PLI Group). All participants underwent a group-based weight management program in addition to four one-on-one sessions (one baseline and three follow-up) with a RD. Group sizes ranged from 7 to 20 participants per group at baseline, with a mean group size of 14 participants. At the three follow-up one-on-one sessions, the RD reviewed the information provided in the summary report (population-based recommendations for the SLI group and DNA-based recommendations for the PLI group; refer to Additional files 2 and 3, respectively, for sample reports). One-on-one sessions lasted approximately 30 min. The same RD who completed the one-on-one sessions was also the lead trained lifestyle coach for the GLB™ Program group sessions. This allowed for optimization of intervention reliability in all group and one-on-one sessions. These sessions were highly standardized as outlined in Additional file 4. No additional healthcare professionals above and beyond standard practice were hired to run the intervention at the EEFHT. Interventions took place between May 2017 and September 2019.

##### SLI group meetings (control group)

The standard GLB™ Program curriculum involves group-based education on a sustainable healthy lifestyle and a moderately-low-fat, calorie-controlled nutrition plan as further detailed elsewhere [52]. Standard GLB™ group sessions were 1 h long. The EEFHT expanded the eligibility criteria for this program and offered it to adults with a BMI ≥25 kg/m^2^. In addition to the standard GLB™ Program, participants were provided with an extra 1.5 h group session (the first session), where they were given an overview of population-based information and recommendations for calories, protein, total fat, saturated fat, total unsaturated fat, monounsaturated fat, polyunsaturated fat, meal/snack timing, and physical activity. This information is further detailed in Additional file 2. Upon completion of the 12-month study, participants in the SLI group were given the results of their lifestyle genomics test if they were interested in receiving it.

##### PLI group meetings (intervention group)

The modified GLB™ Program curriculum is outlined in Additional file 4. The modifications allowed participants in this group to follow their DNA-based recommendations, rather than the standard population-based guidelines. For example, if an individual possessed a genetic variant in the FTO gene whereby a moderately high protein diet can enhance weight loss [21], they were given a target for protein, and were taught how to count daily grams of protein (in addition to calories). In comparison, for the standard GLB™ program, every participant was provided with a target for total fat intake and were taught how to count daily fat grams (in addition to calories). Modified GLB™ group sessions were 1 h long. In addition to the modified GLB™ Program, participants were provided with an extra 1.5 h group session (the first session), where they were given an overview of personalized DNA-based information and recommendations for calories, protein, total fat, saturated fat, total unsaturated fat, monounsaturated fat, polyunsaturated fat, meal/snack timing, and physical activity. This information is further detailed in Additional file 3. It should be noted that the genetic intervention is rated to be high-quality based on a recently developed genetic intervention quality assessment tool [11]. The quality assessment is outlined in Additional file 5.

#### Follow-up data collection

Similar to baseline data collection, follow-up data collection involved a combination of in-person, online and telephone data collection methods. All participants were invited to complete follow-up data collection, regardless of their compliance to their intervention’s lifestyle recommendation. Complete follow-up data included: BMI, 3-day food records, the CDHQII past-month online food frequency questionnaire, BIA, a past-week physical activity recall, a follow-up demographic survey and medication list, and a TPB questionnaire. Further details of these measures are indicated above in section 4.6. The TPB questionnaire was administered once at each follow-up time point during the one-on-one in-person sessions. In addition, at the 12-month follow-up, participants were asked one open-ended question: *How has your life changed since you started participating in this program/study (if at all)?* Follow-up data collection commenced in August 2017 and is ongoing until September 2019.

#### Statistical analysis plan

We plan to use SPSS Version 23.0 to conduct all statistical analyses, and the data will be analyzed on an intention-to-treat basis. Generalized linear mixed-effects models will be used to test between group differences from baseline to each follow-up period for each outcome indicator. If significant mean differences are detected, a Tukey’s post hoc test will be used to compare differences by group. General linear regression models will be used to assess interactions between a given genotype of interest and dietary component of interest on BMI and body composition. General linear regression models will further be used to assess interactions between TPB components, study group, and anthropometric measures of weight and body composition. No interim analyses will be completed.

#### Outcomes

The primary outcome in this study is change in percent body fat. Secondary outcome measures include changes in: dietary intake (calories, fat, protein, carbohydrates, unsaturated fat including mono- and poly-unsaturated fat, saturated fat, and sodium), physical activity, attitudes, subjective norms, behavioural control, intention to make lifestyle changes, weight and BMI.

#### Dissemination

We plan to disseminate the findings from this trial through: a community presentation to the participants involved in the study, presentations at relevant conferences for researchers and healthcare professionals, as well as in peer-reviewed publications.

## Discussion

The overarching aim of this study is to determine if patients have improved health and lifestyle outcomes with the provision of DNA-based lifestyle information and recommendations, compared to the provision of standard, population-based lifestyle advice. Furthermore, it aims to test the aforementioned hypotheses, based on lifestyle genomics weight management advice available to consumers globally through commercial genetic testing. This highlights the pragmatic nature of this trial, and optimizes the potential for knowledge translation on a global-scale.

The NOW Trial protocol differs from previous research in that it was designed pragmatically, using a knowledge translation approach. Furthermore, the NOW Trial aims to compare a DNA-based lifestyle change program to the gold standard, population-based lifestyle change program (the GLB™ Program), while considering and accounting for major confounding factors of behaviour change. It is also the first lifestyle genomics weight management and behaviour change study to incorporate the TPB into the study design; this may help target a sub-set of the population that may benefit most from genetic testing for weight management. This trial is also unique because the genetic information was presented to participants in a group setting, thus demonstrating the feasibility of this more efficient approach to the delivery of genetic information.

Pragmatic clinical trials are distinguished by their focus on informing clinical practice rather than confirming a physiological or clinical hypothesis. Notably, pragmatic trials help to inform real-world research questions that are applicable to broad patient groups [53]. Given the novel and pragmatic nature of the study, the NOW Trial provides several original contributions to the literature. Overall, the NOW Trial will provide important, innovative health knowledge relevant to researchers, academia, consumers, the genetic testing industry, clinicians and public health authorities.

## Additional Files

Additional file 1: Proposed Extended CONSORT Checklist of Items for Reporting Pragmatic Trials [42]. Checklist of items to include in a pragmatic RCT (DOCX 18 kb)

Additional file 2: Sample Report for Standard Lifestyle Intervention (Control Group). Sample population-based lifestyle recommendations and information provided to the control group (DOCX 13 kb)

Additional file 3: Sample Report for Personalized Lifestyle Intervention. Sample genetic-based lifestyle recommendations and information provided to the lifestyle genomics intervention group (DOCX 14 kb)

Additional file 4: GLB™ Program [52]/NOW Trial Curriculum and Modifications for Genetic Testing Intervention Groups. Legend for Additional file 4. 1. The physical activity goal and references to fat grams were verbally modified in the “To Do” lists at the end of sessions. Participants were reminded about how response to different diets and physical activity for weight loss differ from person to person. Based on their personalized genetic report, participants were advised and taught how to reach their personal nutrition and physical activity goals. This modification occurred throughout the GLB™ Program’s “To Do” lists and is not included in Additional file 4. 2. The GLB™ Curriculum begins in class 2. Class 1 allows for an overview of nutrition and physical activity guidelines either based on [1] the Acceptable Macronutrient Distribution Ranges and population-based health information and recommendations or [2] genetic-based information and recommendations. Refer to Additional files 2 and 3 for sample reports provided in class 1. 3. Participants were informed about how the program is typically used for individuals with pre-diabetes, since our population consisted of overweight/obese adults who may or may not have pre-diabetes or type 2 diabetes. (DOCX 19 kb)

Additional file 5: Quality Assessment Tool for Genetic Interventions [11]. Legend for Additional file 5. *CD, cannot determine; NR, not reported; NA, not applicable (DOCX 13 kb)

## Abbreviations

BIA: bioelectrical impedance analysis
BMI: body mass index
CDHQII: Canadian Diet History Questionnaire II
EEFHT: East Elgin Family Health Team
GLB™: Group Lifestyle Balance™
PLI: personalized lifestyle intervention
RD: registered dietitian
SLI: standard lifestyle intervention
The NOW Trial: The Nutrigenomics, Overweight/Obesity and Weight Management Trial
TPB: Theory of Planned Behaviour
