# Supplementary material for: Study protocol of a pragmatic randomized controlled trial incorporated into the Group Lifestyle Balance™ program: the nutrigenomics, overweight/obesity and weight management trial (the NOW trial)
Source: BMC Public Health. 2019 Mar 15;19:310. doi: 10.1186/s12889-019-6621-8 (PMC6419841; doi:10.1186/s12889-019-6621-8)
Supplement: Supplementary file 2 — Sample Report for Standard Lifestyle Intervention (Control Group). Sample population-based lifestyle recommendations and information provided to the control group (DOCX 13 kb) [file 12889_2019_6621_MOESM2_ESM.docx]

**Supplement 2**

| **Lifestyle Component** | **Population-Based Recommendations** |
| --- | --- |
| Calories | Aim for a 500 calorie deficit per day for weight loss. |
| Protein | Consume 10-35% of calories from protein. |
| Total Fat | Consume 20-35% of calories from fat. |
| Saturated Fat | Limit your saturated fat intake to less than 10% of total calories. |
| Unsaturated Fat | Consume a balance of monounsaturated and polyunsaturated fat to meet your total fat needs. |
| Monounsaturated Fat | Consume a balance of monounsaturated and polyunsaturated fat to meet your total fat needs. |
| Polyunsaturated Fat |  |
| Sodium | Consume less than 2300 mg sodium daily. |
| Eating Between Meals | Do not go longer than six hours without eating throughout the day. Ensure snacks and meals are calorie-controlled. |
| Physical Activity | Aim for 150 minutes/week of physical activity with muscle strengthening activities at least 2 days/week. |
| Endurance | Find an endurance-based activity that you enjoy – meet the physical activity guidelines stated above. |
| Strength and Power | Find a strength/power based activity that you enjoy – meet the physical activity guidelines stated above. |
