# Supplementary material for: Study protocol of a pragmatic randomized controlled trial incorporated into the Group Lifestyle Balance™ program: the nutrigenomics, overweight/obesity and weight management trial (the NOW trial)
Source: BMC Public Health. 2019 Mar 15;19:310. doi: 10.1186/s12889-019-6621-8 (PMC6419841; doi:10.1186/s12889-019-6621-8)
Supplement: Supplementary file 3 — Sample Report for Personalized Lifestyle Intervention. Sample genetic-based lifestyle recommendations and information provided to the lifestyle genomics intervention group (DOCX 14 kb) [file 12889_2019_6621_MOESM3_ESM.docx]

**Supplement 3**

| **Lifestyle Component** | **Gene(s), rs number(s)** | **Your Genetic Variant** | **Your Risk/**  **Response** | **DNA-Based Recommendations and Implication** |
| --- | --- | --- | --- | --- |
| Calories | UCP1, rs1800592 | AA | Typical | Your resting metabolism is typical. Aim for a 500 calorie deficit per day for weight loss. |
| Protein | FTO, rs9939609 | AA | Enhanced | You can enhance your weight loss if you consume 25-35% of calories from protein. |
| Total Fat | TCF7L2, rs7903146 | TC | Typical | Consume 20-35% of calories from fat. |
| Saturated Fat | APOA2, rs5082 | CC | Enhanced | You can enhance your weight loss if you consume less than 10% of calories from saturated fat. |
| Unsaturated Fat | FTO, rs9939609 | AA | Enhanced | You can enhance your weight loss if you limit your intake of saturated fat to less than 10% of calories and consume at least 5% of calories from polyunsaturated fat. |
| Monounsaturated Fat | PPARg2, rs1801282 | CC | Typical | Consume a balance of monounsaturated and polyunsaturated fat to meet your total dietary fat intake goal. |
| Sodium | ACE, rs4343 | GG | Typical | Limit your sodium intake to less than 2300 mg per day for heart health. |
| Eating Between Meals | MC4R, rs17782313 | TT | Typical | You have a typical likelihood of eating between meals. Do not go longer than six hours without eating. |
| Physical Activity | FTO, rs9939609 | AA | Enhanced | You can enhance your weight loss if you complete at least 30-60 minutes/day of cardio activity, 6 days/week and muscle-strengthening activities at least 2 days/week. |
| Endurance | ADRB, rs4994 | TT | Typical | You have a typical genetic predisposition to excel in endurance-based physical activity. |
|  | NRF2, rs12594956 | CA |  |  |
|  | GSTP1, rs1695 | AA |  |  |
|  | NFIA-AS2, rs1572312 | CC |  |  |
| Strength and Power | ACTN3, rs1815739 | TC | Enhanced | You have an enhanced genetic predisposition to excel in strength and power based physical activity. |
|  |  |  |  |  |
| **Participant Number** | **X** |  |  |  |
