# Supplementary material for: Study protocol of a pragmatic randomized controlled trial incorporated into the Group Lifestyle Balance™ program: the nutrigenomics, overweight/obesity and weight management trial (the NOW trial)
Source: BMC Public Health. 2019 Mar 15;19:310. doi: 10.1186/s12889-019-6621-8 (PMC6419841; doi:10.1186/s12889-019-6621-8)
Supplement: Supplementary file 4 — GLB™ Program [52]/NOW Trial Curriculum and Modifications for Genetic Testing Intervention Groups. Legend for Additional file 4. 1. The physical activity goal and references to fat grams were verbally modified in the “To Do” lists at the end of sessions. Participants were reminded about how response to different diets and physical activity for weight loss differ from person to person. Based on their personalized genetic report, participants were advised and taught how to reach their personal nutrition and physical activity goals. This modification occurred throughout the GLB™ Program’s “To Do” lists and is not included in Additional file 4. 2. The GLB™ Curriculum begins in class 2. Class 1 allows for an overview of nutrition and physical activity guidelines either based on [1] the Acceptable Macronutrient Distribution Ranges and population-based health information and recommendations or [2] genetic-based information and recommendations. Refer to Additional files 2 and 3 for sample reports provided in class 1. 3. Participants were informed about how the program is typically used for individuals with pre-diabetes, since our population consisted of overweight/obese adults who may or may not have pre-diabetes or type 2 diabetes. (DOCX 19 kb) [file 12889_2019_6621_MOESM4_ESM.docx]

**Supplement 4**

| **Class Number** | **Class Topic** | **Modifications for Genetic Testing Intervention Groups^1^** |
| --- | --- | --- |
| 1 | General Overview of Nutrition and Physical Activity Targets^2^ | - Genetic information and recommendations provided to participants |
| 2 | Welcome to the Group Lifestyle Balance™ Program^3^ | - The physical activity goal was verbally modified whereby participants were asked to refer to their personalized physical activity goals from their genetic report. |
| 3 | Be a Calorie Detective | - Any reference made to counting fat grams was verbally modified. Participants were reminded about how response to different diets for weight loss differ from person to person. Based on their personalized genetic report, participants were advised and taught how to count a nutrient that would benefit their personal weight loss (i.e. some counted protein, others counted saturated fat, and/or total fat, etc). - The calorie goals remained the same, but participants with the “diminished” result in their genetic report for calories were advised to be especially mindful of meeting their calorie goal, and were advised to aim for a 650 kcal deficit to lose 1 lb per week. |
| 4 | Healthy Eating | - When reference was made to a nutrient included in the genetic report, participants were instructed to refer back to their genetic report to recall how this nutrient might be particularly important to them. The information in the genetic reports was then reviewed. |
| 5 | Move Those Muscles | - The physical activity goal was verbally modified whereby participants were asked to refer to their personalized physical activity goals from their genetic report. - Genetic predisposition to excel in endurance and/or strengthening activities (outlined in the genetic report) was reviewed. |
| 6 | Tip the Calorie Balance | - For the daily calorie deficit for weight loss, participants were advised to refer to their genetic report to determine if they should aim for a 500 kcal deficit/day or a 650 kcal deficit/day. - When reference was made to a nutrient included in the genetic report, participants were instructed to refer back to their genetic report to recall how this nutrient might be particularly important to them. The information in the genetic reports was then reviewed. |
| 7 | Take Charge of What’s Around You | - No^1^ modifications were made. Some participants discussed components of their genetic report. |
| 8 | Problem Solving | - No^1^ modifications were made. Some participants discussed components of their genetic report. |
| 9 | Step Up Your Physical Activity Plan | - The physical activity goal was verbally modified whereby participants were asked to refer to their personalized physical activity goals from their genetic report. - Genetic predisposition to excel in endurance and/or strengthening activities (outlined in the genetic report) was reviewed. - Participants with the “enhanced” weight loss response to physical activity (from the genetic report), were advised to continue working up to 30-60 mins/day, 6 days/week of moderate intensity physical activity. |
| 10 | Manage Slips & Self-Defeating Thoughts | - The step goal was verbally modified for individuals with the “enhanced” weight loss response to physical activity; these individuals were advised to aim for 10,000 steps/day. - When reference was made to a nutrient included in the genetic report, participants were instructed to refer back to their genetic report to recall how this nutrient might be particularly important to them. The information in the genetic reports was then reviewed. |
| 11 | Four Keys to Healthy Eating Out | - No^1^ modifications were made. Some participants discussed components of their genetic report. |
| 12 | Make Social Cues Work for You | - No^1^ modifications were made. Some participants discussed components of their genetic report. |
| 13 | Ways to Stay Motivated | - The physical activity goal was verbally modified whereby participants were asked to refer to their personalized physical activity goals from their genetic report. - The step goal was verbally modified for individuals with the “enhanced” weight loss response to physical activity; these individuals were advised to aim for 10,000 steps/day. |
| 14 | Strengthen Your Physical Activity Plan | - The physical activity goal was verbally modified whereby participants were asked to refer to their personalized physical activity goals from their genetic report. - The step goal was verbally modified for individuals with the “enhanced” weight loss response to physical activity; these individuals were advised to aim for 10,000 steps/day. Genetic predisposition to excel in endurance and/or strengthening activities (outlined in the genetic report) was reviewed. |
| 15 | Take Charge of Your Lifestyle | - Reference made to fat grams was verbally modified. Participants were reminded about how response to different diets for weight loss differ from person to person. Based on their personalized genetic report, participants were advised to count a nutrient that would benefit their personal weight loss (i.e. some counted protein, others counted saturated fat, and/or total fat, etc). |
| 16 | Mindful Eating, Mindful Movement | - No^1^ modifications were made. Some participants discussed components of their genetic report. |
| 17 | Manage Your Stress | - No^1^ modifications were made. Some participants discussed components of their genetic report. |
| 18 | Sit Less for Your Health | - No^1^ modifications were made. Some participants discussed components of their genetic report. |
| 19 | More Volume, Fewer Calories | - When reference was made to a nutrient included in the genetic report, participants were instructed to refer back to their genetic report to recall how this nutrient might be particularly important to them. The information in the genetic reports was then reviewed. |
| 20 | Stay Active | - No^1^ modifications were made. Some participants discussed components of their genetic report. |
| 21 | Balance Your Thoughts | - No^1^ modifications were made. Some participants discussed components of their genetic report. |
| 22 | Heart Health | - When reference was made to a nutrient included in the genetic report, participants were instructed to refer back to their genetic report to recall how this nutrient might be particularly important to them. The information in the genetic reports was then reviewed. - The physical activity goal was verbally modified whereby participants were asked to refer to their personalized physical activity goals from their genetic report. - The step goal was verbally modified for individuals with the “enhanced” weight loss response to physical activity; these individuals were advised to aim for 10,000 steps/day. Genetic predisposition to excel in endurance and/or strengthening activities (outlined in the genetic report) was reviewed. |
| 23 | Look Back & Look Forward | - The physical activity goal was verbally modified whereby participants were asked to refer to their personalized physical activity goals from their genetic report. - The step goal was verbally modified for individuals with the “enhanced” weight loss response to physical activity; these individuals were advised to aim for 10,000 steps/day. Genetic predisposition to excel in endurance and/or strengthening activities (outlined in the genetic report) was reviewed. |
