# Supplementary material for: Study protocol of a pragmatic randomized controlled trial incorporated into the Group Lifestyle Balance™ program: the nutrigenomics, overweight/obesity and weight management trial (the NOW trial)
Source: BMC Public Health. 2019 Mar 15;19:310. doi: 10.1186/s12889-019-6621-8 (PMC6419841; doi:10.1186/s12889-019-6621-8)
Supplement: Supplementary file 5 — Quality Assessment Tool for Genetic Interventions [11]. Legend for Additional file 5. *CD, cannot determine; NR, not reported; NA, not applicable (DOCX 13 kb) [file 12889_2019_6621_MOESM5_ESM.docx]

**Supplement 5**

| **Criteria** | **Yes** | **No** | **Other (CD, NR, NA)*** |
| --- | --- | --- | --- |
| 1. Were the results of the genetic test interpreted and explained by a trained healthcare professional? | 🗸 |  |  |
| 2. Was a copy of the genetic testing report provided to the participants? | 🗸 |  |  |
| 3. Were the results of the genetic test communicated to participants on more than one occasion (i.e. was there follow-up provided after the initial communication of the results)? | 🗸 |  |  |
| 4. Were the results provided in the report, or discussed in the genetic counselling session actionable (i.e. did the report contain specific recommendations or did the genetic counsellor communicate specific recommendations)? | 🗸 |  |  |
| 5. Were the participants provided with an opportunity to ask questions about their results? | 🗸 |  |  |

Other Comments: N/A ______________________________________________________________

**Overall Rating** (Good, Fair, Poor; if Poor state reasons): **Good**
